# Supplementary material for: Researchers’ perspective of real-world impact from UK public health research: A qualitative study
Source: PLoS One. 2022 Jun 27;17(6):e0268675. doi: 10.1371/journal.pone.0268675 (PMC9236254; doi:10.1371/journal.pone.0268675)
Supplement: S1 File — Semi-structures questions that were asked of participants during telephone interviews. (DOCX) [file pone.0268675.s001.docx]

# Lakin K, Meadmore K, Recio Saucedo A, Baker G, Worswick L, Thomas S. Researchers’ perspective of real-world impact from UK public health research: a qualitative study. PLoS One. 2022 10.1371/journal.pone.0268675.

**S1 File. Interview Schedule**

**Mapping the impact of National Institute for Health Research Evaluation, Studies and Trials Coordinating Centre (NETSCC) funded research**

(Researchers have provided participants with the participant information form and consent form in advance of the interview).

Consent process is undertaken and recorded in a separate audio file from this interview recording.

Thank you for your time today.

Do you have any questions before we start the recording for the interview?

**Could you tell us a bit about <research project> and your role with the project?**

**How did you become involved?**

**What was the rationale behind the project?**

**What impact(s) did you hope the project have initially?**

**What in your view was or could be the benefit or impact of the research project e.g**

**Health impacts?**

**Did the research influence a guideline? If so at what level?**

**Policy influences?**

**Social impact or societal change (change in perception/understandings of health-related behaviour/ change in practice)**

**Changes in education and training?**

**Changes in industry?**

**Financial or economic impact?**

**Academic?**

**No impact?**

**Anything else?**

1. **If impacts have been achieved from the research, who do you believe has benefited from those impacts?**
   1. **Which groups of people have benefitted eg. Patient groups, staff groups, other groups in the community? etc**
   2. **In what way?**
   3. **How widespread was the impact? Eg did it affect large groups/multiple groups?**
   4. **Also geographical spread?**
   5. **Is the impact still ongoing? Is there potential for further impact? If so, how might this be achieved?**

**What do you think was the most important/useful impact? Why?**

1. **Which stakeholders were involved in facilitating the impact for this research?**
   1. **For example, was the media involved, or policymakers, community groups, charities, local authority, lobby groups etc?**
   2. **How did they facilitate the impact?**

**How do you know the project had the impact you describe? What evidence is there? E.g.**

**Policy documents**

**Reports**

**Websites**

**Commentary or feedback from stakeholders**

**Social media**

**local news media**

**Other**

**If there was no impact to report, why do you think this is the case?**

**What methods did you use to promote/share your findings? For example what engagement activities?**

1. **Were there any subsequent activities/follow up after the end of the project? e.g.**
   1. **Did you follow up with beneficiaries?**
   2. **Did you continue to contribute to committees/documents etc. arising from the research?**
2. **What do you think was the most effective way in which impact was achieved?**
   1. **And did you encounter any problems or barriers to pursuing the intended impact?**
3. **Is there anyone else involved in the research who you think we should speak to?**
4. **Is there anything else we should consider in order to understand the ways in which impact is achieved?**
