# Supplementary material for: Researchers’ perspective of real-world impact from UK public health research: A qualitative study
Source: PLoS One. 2022 Jun 27;17(6):e0268675. doi: 10.1371/journal.pone.0268675 (PMC9236254; doi:10.1371/journal.pone.0268675)
Supplement: Attachment — Submitted filename: Lakin et al Response to reviewers.docx [file pone.0268675.s002.docx]

Dear Chaisiri Angkurawaranon,

**Researchers’ perspective of real-world impact from UK public health research: a qualitative study**

Kay E. Lakin, Katie Meadmore, Alejandra Recio Saucedo, Genevieve Baker, Louise Worswick, Sarah E. Thomas

On behalf on my co-authors, we wish to thank you and your reviewers for such positive reviews on this manuscript. We have revised the manuscript based on the reviewers’ comments and have attached a point-by–point response (indented in bold) to each reviewer comment. We hope this revised and improved manuscript is now suitable for publication in PLOS ONE.

As requested, Please find below our updated Competing Interests Statement and Data Sharing statement:

All authors worked for the NIHR Evaluation, Trials and Studies Coordinating Centre (NETSCC) at the time of this study. However, no author was involved in any aspect of the research management process or any funding recommendations. This does not alter our adherence to PLOS ONE policies on sharing data and materials.

As per the ethical approvals in place for this project and to maintain the confidentiality of the interviewees, whole interviews will not be shared. A sample of relevant quotes are made available in the manuscript under each theme. We are happy to consider requests for additional data, if required. Requests should be made to Kay.Lakin@nihr.ac.uk

Kind regards,

Kay Lakin

**Response to Editors and reviewers**

Journal Requirements:

When submitting your revision, we need you to address these additional requirements.

1. Please ensure that your manuscript meets PLOS ONE's style requirements, including those for file naming. The PLOS ONE style templates can be found at 
<https://journals.plos.org/plosone/s/file?id=wjVg/PLOSOne_formatting_sample_main_body.pdf> and 
<https://journals.plos.org/plosone/s/file?id=ba62/PLOSOne_formatting_sample_title_authors_affiliations.pdf>.

**Apologies that the manuscript did not meet the PLOS ONE style requirements. We have revisited the guidance and revised the document accordingly.**

2. We note that you obtained audio consent. Please state in the Methods:
- Why written consent could not be obtained
- Whether the Institutional Review Board (IRB) approved use of oral consent
For more information, please see our guidelines for human subjects research: <https://journals.plos.org/plosone/s/submission-guidelines#loc-human-subjects-research>

**Verbal consent was obtained as this was felt to be the most practical approach to getting informed consent when all interviews were planned to be conducted over the phone. This approach was approved by the University of Southampton, Faculty of Medicine ethics committee. This justification has been added to the methods section**

3. In your Methods section, please provide additional information about the participant recruitment method and the demographic details of your participants. Please ensure you have provided sufficient details to replicate the analyses such as descriptions of where participants were recruited and where the research took place.

**Additional recruitment information has been added and the recruitment process has been**	**revised to make it clearer how participants were recruited and where the research took**	**place. Please note that demographic data was not collected from participants in order to**	**maintain confidentiality and chance of identification given the small potential sample.**

4. Please include a copy of the interview guide used in the study, in both the original language and English, as Supporting Information, or include a citation if it has been published previously.

**A copy of the interview has been included as a supplementary file (File S1 – Interview Framework)**

5. Thank you for stating the following in the Competing Interests section: "All authors worked for the NIHR Evaluation, Trials and Studies Coordinating Centre (NETSCC) at the time of this study. However, no author was involved in any aspect of the research management process or any funding recommendations."

Please confirm that this does not alter your adherence to all PLOS ONE policies on sharing data and materials, by including the following statement: "This does not alter our adherence to  PLOS ONE policies on sharing data and materials.” (as detailed online in our guide for authors <http://journals.plos.org/plosone/s/competing-interests>).  If there are restrictions on sharing of data and/or materials, please state these. Please note that we cannot proceed with consideration of your article until this information has been declared.

Please include your updated Competing Interests statement in your cover letter; we will change the online submission form on your behalf.

**We have updated our Competing Interests statement in the cover letter to include the line: “This does not alter our adherence to PLOS ONE policies on sharing data and materials.”**

6. We note that you have indicated that data from this study are available upon request. PLOS only allows data to be available upon request if there are legal or ethical restrictions on sharing data publicly. For more information on unacceptable data access restrictions, please see <http://journals.plos.org/plosone/s/data-availability#loc-unacceptable-data-access-restrictions>.

In your revised cover letter, please address the following prompts:

a) If there are ethical or legal restrictions on sharing a de-identified data set, please explain them in detail (e.g., data contain potentially sensitive information, data are owned by a third-party organization, etc.) and who has imposed them (e.g., an ethics committee). Please also provide contact information for a data access committee, ethics committee, or other institutional body to which data requests may be sent.

b) If there are no restrictions, please upload the minimal anonymized data set necessary to replicate your study findings as either Supporting Information files or to a stable, public repository and provide us with the relevant URLs, DOIs, or accession numbers. For a list of acceptable repositories, please see <http://journals.plos.org/plosone/s/data-availability#loc-recommended-repositories>.

We will update your Data Availability statement on your behalf to reflect the information you provide.

**We have provided updated our Data Availability statement in the cover letter to state: As per the ethical approvals in place for this project, to maintain the confidentiality of the interviewees whole interviews will not be shared. A sample of relevant quotes are made available in the manuscript and we are happy to consider requests for additional quotes if required. Requests should be made to K.Lakin@nihr.ac.uk**

7. Your abstract cannot contain citations. Please only include citations in the body text of the manuscript, and ensure that they remain in ascending numerical order on first mention.

**We can confirm that the abstract does not contain citations**

8. We note that you have referenced "Lakin K, Baker G, Thomas S, Worswick L, Dorling H. NIHR Public Health Research Programme: Exploring the influence of research on policy & practice" which has currently not yet been accepted for publication. Please remove this from your References and amend this to state in the body of your manuscript: "Lakin K, Baker G, Thomas S, Worswick L, Dorling H. NIHR Public Health Research Programme: Exploring the influence of research on policy & practice. Unpublished internal report; 2018." as detailed online in our guide for authors <http://journals.plos.org/plosone/s/submission-guidelines#loc-reference-style>.

**This has been amended as requested**


Reviewers' comments:

Reviewer's Responses to Questions

**Comments to the Author**

1. Is the manuscript technically sound, and do the data support the conclusions?

The manuscript must describe a technically sound piece of scientific research with data that supports the conclusions. Experiments must have been conducted rigorously, with appropriate controls, replication, and sample sizes. The conclusions must be drawn appropriately based on the data presented.

Reviewer #1: Yes

Reviewer #2: Partly

2. Has the statistical analysis been performed appropriately and rigorously?

Reviewer #1: Yes

Reviewer #2: N/A

3. Have the authors made all data underlying the findings in their manuscript fully available?

The [PLOS Data policy](http://www.plosone.org/static/policies.action#sharing) requires authors to make all data underlying the findings described in their manuscript fully available without restriction, with rare exception (please refer to the Data Availability Statement in the manuscript PDF file). The data should be provided as part of the manuscript or its supporting information, or deposited to a public repository. For example, in addition to summary statistics, the data points behind means, medians and variance measures should be available. If there are restrictions on publicly sharing data—e.g. participant privacy or use of data from a third party—those must be specified.

Reviewer #1: Yes

Reviewer #2: Yes

4. Is the manuscript presented in an intelligible fashion and written in standard English?

PLOS ONE does not copyedit accepted manuscripts, so the language in submitted articles must be clear, correct, and unambiguous. Any typographical or grammatical errors should be corrected at revision, so please note any specific errors here.

Reviewer #1: Yes

Reviewer #2: Yes

5. Review Comments to the Author

Please use the space provided to explain your answers to the questions above. You may also include additional comments for the author, including concerns about dual publication, research ethics, or publication ethics. (Please upload your review as an attachment if it exceeds 20,000 characters)

Reviewer #1: There are no major suggestions as such. The manuscript is well written and meets the standard criteria and has passed the COREQ checklist successfully meeting the standards and encompassing all the necessary aspects. However, it is advised that the manuscript is structured accordingly to the criteria highlighted by the journal, although very few minor adjustments are required and correction of few grammatical errors. A professional editing is also advised for typos etc.

**We thank the reviewer for these positive comments. We have revisited the manuscript guidelines and revised the manuscript accordingly. We have also had the manuscript proof-read before resubmission.**

Reviewer #2: Nicely done empirical study - I have no major criticisms of that aspect. but it's under-theorised. I think given the HUGE knowledge base about knowledge translation and research impact that's accumulated in the past few years, we can't have more papers appearing which just present "three linked themes". You need to theorise this properly, using one or other of the many theoretical frameworks linking research to impact! Personally I'd use either the CIHR framework or Jo Rycroft-Malone's realist evaluation framework (might be diplomatic - she's now head of HS&DR!). This will mean a major re-write but I think worth it. My team reviewed the different impact frameworks a few years ago - there may be better ones out there now.

Trish Greenhalgh

Canadian Academy of Health Sciences: Making an Impact, A Preferred Framework and Indicators to Measure Returns on Investment in Health Research. Downloadable from <http://www.cahs-acss.ca/wp-content/> uploads/2011/09/ROI_FullReport.pdf. Ottawa: CAHS; 2009.

Rycroft-Malone J, Burton C, Wilkinson J, Harvey G, McCormack B, Baker R,
Dopson S, Graham I, Staniszewska S, Thompson C et al: Health Services and Delivery Research. In: Collective action for knowledge mobilisation: a realist evaluation of the Collaborations for Leadership in Applied Health Research and Care. Volume 3, edn. Southampton (UK): NIHR Journals Library.; 2015: 44.

Greenhalgh et al. Research impact - a narrative review. BMC Medicine (2016) 14:78

**We thank Professor Greenhalgh for her comments. We accept that the paper did not explicitly reference the theory behind the thematic framework. The interview questions themselves were informed by the Payback Framework. As such, we have continued to use this theory to link to our findings. We have added a diagram (fig 1) to show how the findings from this study support and map onto the Payback Framework for PH research and have revised the manuscript throughout to reflect this. We agree that this now significantly strengthens the findings of the paper.**

6. PLOS authors have the option to publish the peer review history of their article ([what does this mean?](https://journals.plos.org/plosone/s/editorial-and-peer-review-process#loc-peer-review-history)). If published, this will include your full peer review and any attached files.


If you choose “no”, your identity will remain anonymous but your review may still be made public.


**Do you want your identity to be public for this peer review?** For information about this choice, including consent withdrawal, please see our [Privacy Policy](https://www.plos.org/privacy-policy).

Reviewer #1: No

Reviewer #2: **Yes:** Trisha Greenhalgh

[NOTE: If reviewer comments were submitted as an attachment file, they will be attached to this email and accessible via the submission site. Please log into your account, locate the manuscript record, and check for the action link "View Attachments". If this link does not appear, there are no attachment files.]

While revising your submission, please upload your figure files to the Preflight Analysis and Conversion Engine (PACE) digital diagnostic tool, <https://pacev2.apexcovantage.com/>. PACE helps ensure that figures meet PLOS requirements. To use PACE, you must first register as a user. Registration is free. Then, login and navigate to the UPLOAD tab, where you will find detailed instructions on how to use the tool. If you encounter any issues or have any questions when using PACE, please email PLOS at [figures@plos.org](mailto:figures@plos.org). Please note that Supporting Information files do not need this step.
